# Lithium and Bariatric Surgery: A Balancing Act

**DOI:** 10.1192/bjo.2024.653

**Published:** 2024-08-01

**Authors:** Zarina Anwar, Anjali Rajadurai, Diya Dholakia

**Affiliations:** 1 Leicestershire Partnership NHS Trust, Leicester, United Kingdom; 2 University of Leicester, Leicester, United Kingdom

## Abstract

**Aims:**

Patients with severe mental illness (SMI) are at greater risk of poor physical health with higher prevalence of obesity, cardiovascular disease, diabetes and higher premature mortality than the general population. The reasons are complex and interventions are multifaceted. Obesity is highly prevalent in the general population and pharmacological and surgical treatments have become more widely available; however, SMI patients may face barriers accessing these. This case highlights specific factors for consideration in managing a patient on lithium therapy undergoing sleeve gastrectomy to balance the risk of lithium toxicity with risk of relapse. Currently, there is limited clinical experience of managing lithium in this context.

**Methods:**

49 yr old female diagnosed with schizoaffective disorder well-maintained for several years on aripiprazole depot and 800mg lithium carbonate (Priadel) with therapeutic levels in treatment range (0.4–0.8mmol/L). Severe obesity (BMI 41kg/m^2^) despite dietary modifications and metformin trial, and recently diagnosed with diabetes. Family history of cardiovascular disease and diabetic related complications with early mortality were additional factors in her request for bariatric surgery. Multidisciplinary discussion including patient, psychiatrist, mental health pharmacist, specialist bariatric dietician and GP prior, to ensure sharing of relevant information pertinent to re-titration and monitoring of lithium therapy and risks of toxicity and relapse.

**Results:**

Patient underwent sleeve gastrectomy with discontinuation of lithium 72 hours prior to surgery. Stomach pouch capacity reduced to 120ml and advised daily fluid intake 500–1000ml in first two weeks. Lithium therapy re-commenced when fluid intake adequate and renal function within normal limits. Formulation changed to liquid for 6–8 weeks to avoid disruption to the healing line, and the dose gradually re-titrated with close monitoring of serum lithium levels. Stabilised on reduced dose of 400mg Priadel at 3 months with therapeutic levels. At 6 months BMI reduced to 32kg/m^2^, antihypertensive and metformin discontinued and maintained remission of schizoaffective disorder.

**Conclusion:**

Sleeve gastrectomy is an increasingly common procedure to treat obesity, with potential long-term positive physical health outcomes and reduction in mortality which may have a role in addressing health inequalities for SMI patients. Psychiatrists need to be aware of key aspects of bariatric surgery particularly relating to safe and effective prescribing of psychotropic medication including potential change to liquid or orodispersible formulation in the post-operative period, close monitoring of serum lithium levels in the short and medium term due its narrow therapeutic index, and consideration of longer-term dose adjustments due to ongoing weight loss.

